# Automated biomedical hypothesis generation with time-aware hypergraph contrastive learning

**DOI:** 10.1007/s10115-026-02785-3

**Published:** 2026-05-17

**Authors:** Amir Hassan Shariatmadari, Sikun Guo, Nathan C. Sheffield, Aidong Zhang, Kishlay Jha

**Affiliations:** 1 Department of Computer Science, University of Virginia, 85 Engineer’s Way, Charlottesville, Virginia 22903 USA; 2 Department of Genome Sciences, University of Virginia, 1335 Lee Street, Charlottesville, Virginia 22903 USA; 3 Department of Electrical and Computer Engineering, University of Iowa, 4324 Seamans Center for the Engineering Arts and Sciences, Iowa City, Iowa 52242 USA

**Keywords:** Hypergraphs, Temporal graph learning, Hypothesis generation

## Abstract

Research in scientific domains now generates more than a million articles annually, overwhelming researchers and hindering discovery. This surge has sparked interest in biomedical hypothesis generation (HG), which aims to uncover implicit patterns among biomedical concepts. Most existing methods focus on pairwise link prediction, overlooking the complex, multi-concept relationships underlying many breakthroughs. We introduce *HyHG*, a temporal *Hy*pergraph contrastive learning framework for biomedical *H*ypothesis *G*eneration, which redefines hypotheses as hyperedges—sets of co-mentioned concepts in an article. By representing articles as hyperedges and organizing them into a temporal hypergraph, HyHG captures the evolution of scientific ideas over time. A transformer-based architecture learns from historical hyperedge sequences to predict future hyperedges—sets of concepts likely to co-occur in the future literature. To distinguish genuine hypotheses from misleading ones, HyHG employs a time-anchored contrastive loss and hard negative sampling based on minimal edits to real hyperedges. We demonstrate that HyHG achieves state-of-the-art performance on three biomedical datasets. Our code and data are available at: https://github.com/amir-hassan25/Temporal-Hypergraph-Contrastive-Learning.

## Introduction

Research in scientific domain is witnessing a rapid increase in publications, with more than a million new articles appearing annually in the biomedical and life sciences [1]. Although this explosion of knowledge holds immense potential for discovery, it also burdens researchers with the task of parsing vast and rapidly growing literature to formulate novel hypotheses. Thus, the generation of biomedical hypothesis (HG) has emerged as a central task in the extraction of biomedical text [2–4], with the objective of discovering novel connections between biomedical concepts scattered between disciplines. A classic example is the association between Raynaud’s disease and Fish Oils, which was discovered through manual analysis and later validated clinically [5], illustrating HG’s ability to surface overlooked insights.

**Fig. 1:**
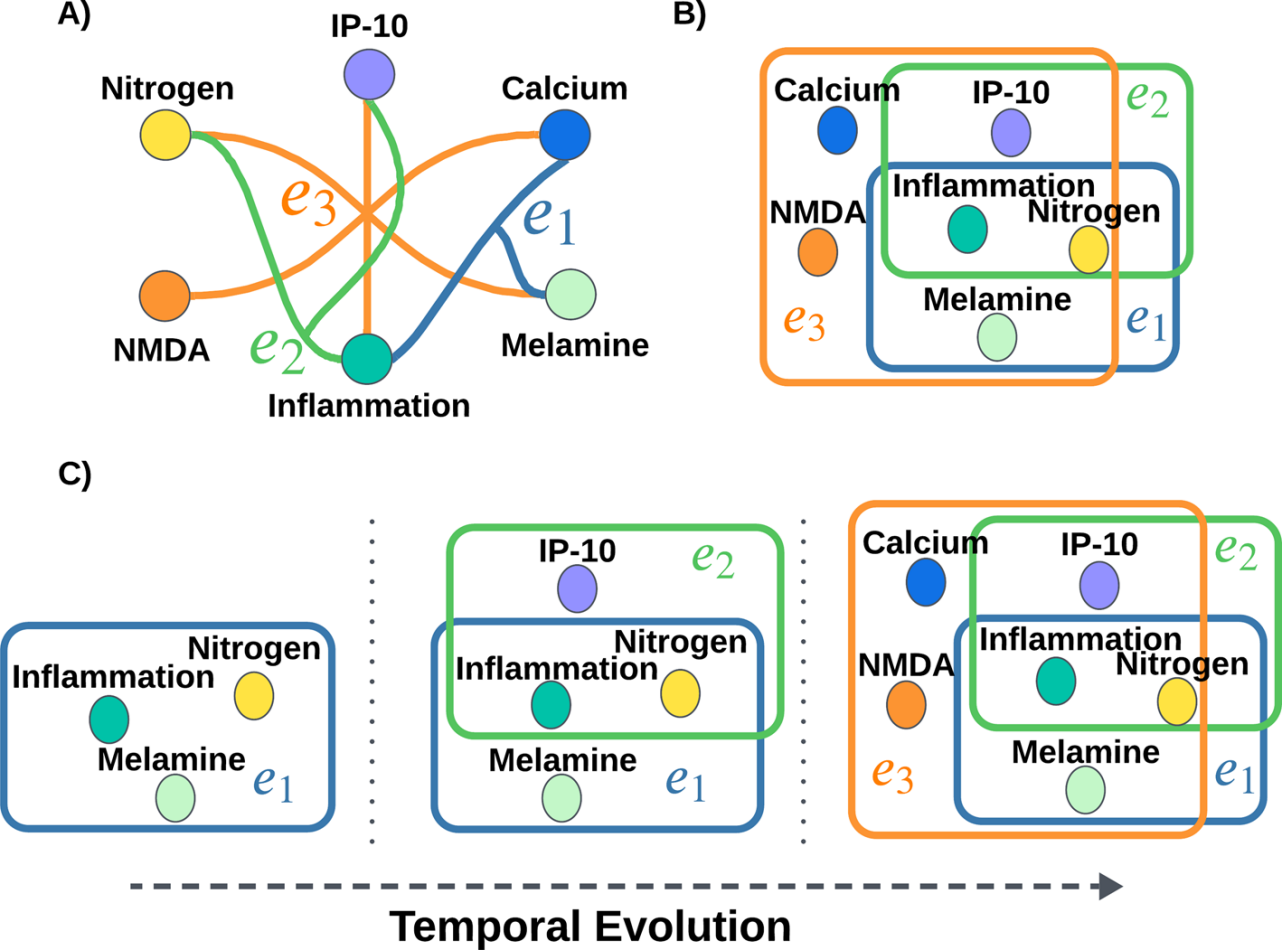
Illustration of hypergraph representations with multi-concept relationships and evolution. **A** Each hyperedge is shown as a colored arc connecting the concepts (nodes) extracted from an article (e.g., $e_1$ in blue, $e_2$ in green, $e_3$ in orange). **B** The same hyperedges are depicted as overlapping rectangles, highlighting how different hyperedges share the common concepts. **C** A temporal view of the hyperedges at different points in time, illustrating how later hyperedges (e.g., $e_3$) build on earlier ones ($e_1$ and $e_2$) by integrating new concepts with established ideas

Most HG methods focus on predicting pairwise links between concepts (e.g., disease–chemical pairs). Early approaches relied on co-occurrence heuristics [5, 6], while more recent work incorporates temporal modeling and learning frameworks to forecast future links [3, 4, 7–11]. However, many biomedical discoveries emerge from complex interactions among multiple concepts, beyond what pairwise models can represent. To capture richer interactions, hypergraphs offer a natural abstraction, with nodes representing biomedical concepts and hyperedges capturing sets of co-mentioned concepts in an article. Figure 1A and B illustrates this hypergraph representation. Advances in hypergraph models have demonstrated strong performance in capturing such higher-order relationships [12–16], achieving success in tasks ranging from node classification to hyperedge prediction [12, 17], and in domains such as metabolic reaction modeling [14] and clinical outcome forecasting [16]. Nevertheless, existing hypergraph methods such as UniGCN, UniGAT [13], CHESHIRE [14], and AHP [18] share key limitations: they either treat hypergraphs as static (focusing on hyperedge prediction in atemporal settings) or, when incorporating negatives, do not combine multi-concept structure with temporal evolution. HyHG addresses these gaps by framing HG as future hyperedge prediction and explicitly modeling how hypotheses evolve over time. Figure 1C illustrates how hyperedges can evolve over time by building upon past ones with new biomedical insights. Such progressions motivate the need for temporal models that can trace and forecast the evolution of multi-concept interactions.

We propose **HyHG**, a temporal hypergraph contrastive learning framework that reframes HG as a future hyperedge prediction task. Each hypothesis is modeled as a hyperedge, representing a coherent group of concepts from an article, rather than as a pairwise link. HyHG aims to forecast which sets of concepts will appear together in future publications, effectively generating new hypotheses grounded in multi-concept structure. HyHG constructs a temporal hypergraph by merging yearly hypergraphs from PubMed articles, capturing how hypotheses evolve. For each target hyperedge, we generate trails of related past hyperedges via a temporally constrained random walk, providing historical context. A transformer-based model with masked attention learns to predict future hyperedges using this structured historical context. To address the challenge of deceptive hypotheses, i.e., those superficially similar but not truly grounded in biomedical progression, HyHG employs hard negative sampling and a time-anchored contrastive loss. Negative samples are crafted via minimal edits to real hyperedges, and the model learns to separate genuine hypotheses from misleading ones based on temporal context. In particular, our contributions can be summarized as:

- We introduce HyHG, the first HG framework to formulate hypothesis generation as future hyperedge prediction, capturing complex multi-concept relationships within publications.
- We propose a temporal hypergraph contrastive learning framework that models evolving trails of ideas and uses a transformer with time-anchored contrastive loss to predict future hypotheses.
- We demonstrate that HyHG achieves state-of-the-art performance across three HG datasets, effectively handling hard negatives and extending static hypergraph models to temporal settings.

The work reported in this paper is an extension of our previous work published at ICDM 2025 [19]. This journal version adds: (i) Algorithm 1 for generating trails of ideas; (ii) Algorithm 2 formalizing the training procedure; (iii) detailed positional encoding derivation in § 3.6.1; (iv) three domain-specific case studies validating HyHG’s ability to rediscover biomedical advancements; (v) analysis sections on robustness (§ 4.7.1) and novelty (§ 4.7.2); (vi) quantitative component contribution analysis in the ablation study (§ 4.3); (vii) dataset difficulty and method progression analysis (§ 4.4) demonstrating HyHG’s 28.3% improvement over temporal baselines; and (viii) method consistency and generalization analysis (§ 4.5) showing 3.6% coefficient of variation across datasets. These additions strengthen the methodology’s clarity, empirical validation, and support the contributions above.

## Related work

Early literature-based discovery demonstrated that latent associations could link disparate biomedical concepts [5]. Swanson’s work and systems like ARROWSMITH [5] pioneered HG by uncovering hidden relationships in public literature, though they relied on simple patterns and heuristics that limited their ability to model the growing complexity of biomedical knowledge.

Recent methods [20–25] improve upon these by modeling temporal dynamics of concept pairs. [4] proposed a temporal positive-unlabeled framework using variational inference to estimate evolving pairwise priors. [7] introduced time-sliced link prediction for forecasting new associations in Alzheimer’s disease knowledge graphs. Other methods extract context-driven subgraphs for improved accuracy and interpretability [26]. Attention-based models, including spatio-temporal transformers [3], temporal attention networks [8, 9], learn embeddings that capture how pairwise relationships evolve over time. To further refine temporal dynamics, recent work has introduced Temporal Self and Interactive Evolution (TSIE) modeling, which utilizes dual-tower transformers to capture both the individual evolution of terms and the interactive evolution of term pairs, demonstrating the value of fine-grained temporal feature learning [27].

While prior methods focus on pairwise links, real-world discoveries often involve multi-concept interactions. Hypergraph Neural Networks (HGNNs) are a class of neural models that learn from hypergraph structures by capturing higher-order interactions among multiple nodes. Within this family, Hypergraph Convolutional Networks (HCNs) form a subclass that generalizes graph convolutions to the hypergraph domain via node-hyperedge aggregation [13]. Notable HCNs include HypergraphConv [12], which propagates features using normalized aggregation; UniGCN and UniGAT [13], which extend GCN and GAT to the hypergraph setting; and CHESHIRE [14], which leverages Chebyshev spectral filters over clique-expanded hypergraphs. Addressing structural limitations in standard hypergraphs, Directed Hypergraph Neural Networks (DHGNN) have recently been proposed to preserve the directionality of hyperedges via an approximate Laplacian, enabling the modeling of asymmetric high-order correlations [28]. Other HGNNs use different mechanisms: CACHE [16] employs self-attention with counterfactual loss for interpretable predictions in EHR data; NHP [15] predicts higher-order patterns using a neural network; and AHP [18] introduces adversarial training to dynamically generate challenging negative hyperedges for more robust hypergraph training.

Although some temporal HGNNs have been explored in domains such as POI recommendation [29] and crime prediction [30], they are domain-specific. More recently, efforts to unify complex graph properties have led to the Heterogeneous Temporal Hypergraph Neural Network (HTHGN), which employs hierarchical attention to model heterogeneity, dynamics, and group interactions simultaneously[31]. However, these approaches are not directly applicable to biomedical HG, which requires modeling evolving, multi-concept hypotheses.

Our work is the first to frame hypothesis generation, typically treated as a pairwise link prediction problem, as a temporal hyperedge prediction task. By modeling each article as a hyperedge and integrating temporal context, our framework captures both multi-concept structure and its evolution for hypothesis generation.

**Fig. 2:**
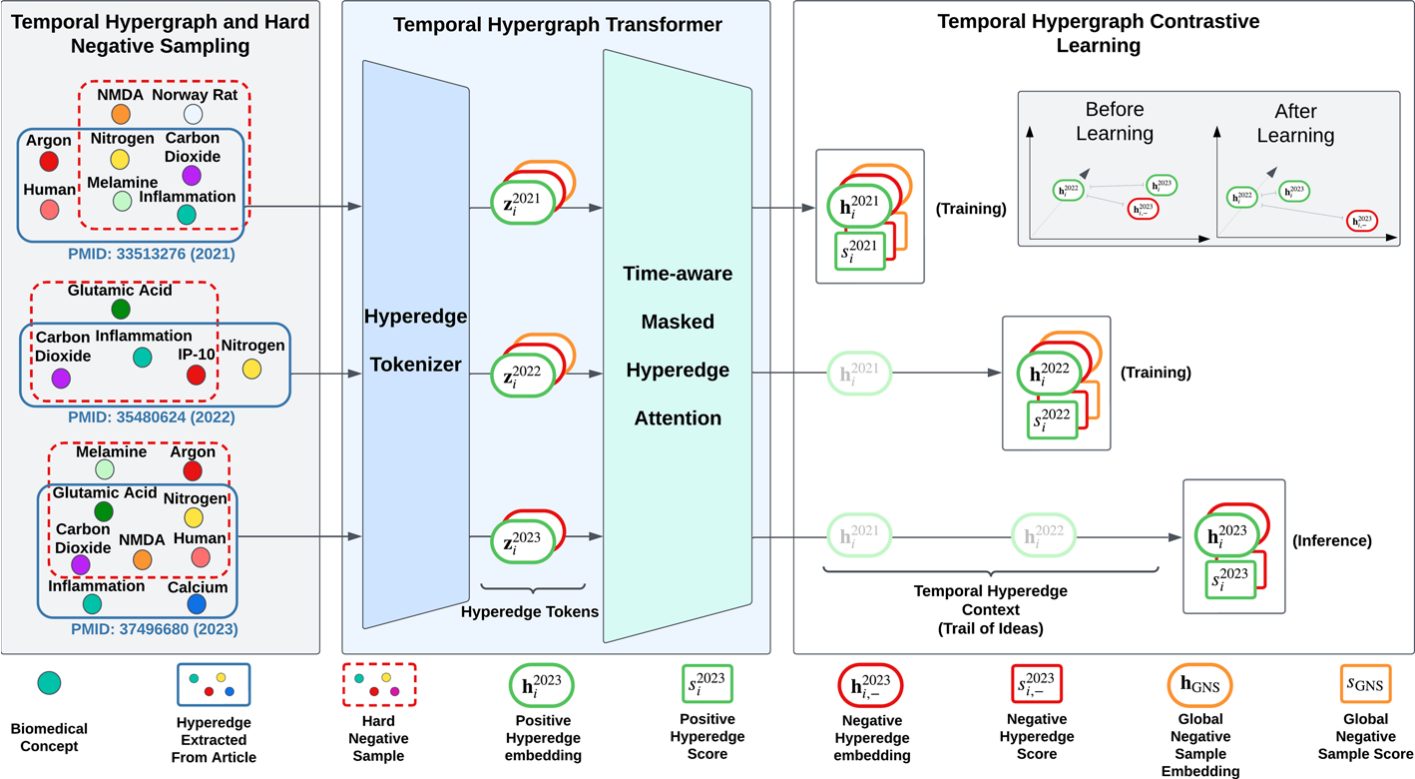
Overview of our proposed framework. The left panel extracts hyperedges of biomedical concepts from PubMed articles (identified by PubMed Identifying Number (PMID)) from various years and generates hard negative samples by marginally modifying real hyperedges. The middle panel represents the Temporal Hypergraph Transformer, where a tokenizer encodes hyperedges, and Time-aware Masked Attention generates contextualized embeddings. The right panel illustrates Temporal Hypergraph Contrastive Learning, where the model sequentially learns from past hyperedges (2021, 2022) to predict a future one (2023), by enforcing separation between positive and deceptive negative hyperedges

## Methodology

We present HyHG, our novel temporal hypergraph contrastive learning framework for hypothesis generation (HG). An overview is illustrated in Fig. 2. First, we construct a temporal hypergraph from biomedical research papers, with each hyperedge representing a set of biomedical concepts extracted from an article. Next, we generate hard negative samples and then simulate idea evolution by linking hyperedges into trails. We then define a prediction objective that leverages these trails to distinguish genuine from fake hyperedges. Finally, a transformer-based architecture integrates local, global, and temporal information to produce contextualized embeddings, with training governed by a combined binary cross-entropy and time-anchored contrastive loss. The following sections detail each component.

### Notations

Following the formulation in [13], we define a hypergraph as $\mathcal {H} = (\mathcal {V}, \mathcal {E})$, where $\mathcal {V}$ is a set of *N* nodes, represented as $\{1, 2, \ldots , N\}$, and $\mathcal {E}$ is a set of *M* hyperedges, $\{e_1, e_2, \ldots , e_M\}$. Each hyperedge $e_i$ is a subset of nodes (i.e., $e_i \subseteq \mathcal {V}$) and for any given node $j \in \mathcal {V}$, the set of hyperedges that are incident to it is denoted by $E_j = \{e \in \mathcal {E} \mid j \in e\}$.

A HCN is designed to operate on static hypergraphs and produce embeddings for hyperedges based on the hypergraph’s structural properties. Its goal is to learn node representations that can be used to generate hyperedge embeddings $\{\textbf{z}_1, \textbf{z}_2, \ldots , \textbf{z}_M\}$, where $\textbf{z}_i \in \mathbb {R}^d$ is a *d*-dimensional embedding for hyperedge $e_i$. The general framework for an HCN involves two phases: message passing and embedding generation. The message passing stage is twofold. First, it aggregates the features of all nodes within a single hyperedge using a permutation-invariant function $\phi _1$ (e.g., mean or summation). For a given hyperedge $e_i$, we compute: $h_i = \phi _1(\{x_j\}_{j\in e_i})$, where $x_j \in \mathbb {R}^d$ represents the *d*-dimension feature vector for node *j*. Second, for each node *j*, we update its representation by aggregating the information from all hyperedges that are incident to it. Specifically, for each hyperedge $e_i \in E_j$, we have already computed a combined feature $h_i$. We then update the node’s feature by applying an aggregation function $\phi _2$ that integrates its original feature $x_j$ with the set of aggregated hyperedge features $\{h_i\}_{e_i \in E_j}$: $\tilde{x}_j = \phi _2(x_j, \{h_i\}_{e_i \in E_j}).$

The way $\phi _1$ and $\phi _2$ are defined determines what type of HCN it is. More details on this are discussed in [13]. After message passing, each node *j*’s features are updated as $\tilde{x}_j$. The hyperedge embedding for $e_i$ is then generated by averaging over the updated features of all nodes in $e_i$ with $\textbf{z}_i = \frac{1}{|e_i|}\sum _{j \in e_i} \tilde{x}_j,$ where $|e_i|$ is the number of nodes in hyperedge $e_i$.

### Temporal hypergraph construction

A temporal hypergraph is defined as a sequence of hypergraphs $\mathcal {H}^{(t)} = (\mathcal {V}^{(t)}, \mathcal {E}^{(t)} )$ over *T* discrete time steps $t \in \{0,1, \ldots , T\}$. In this formulation, $\mathcal {V}^{(t)}$ is the set of nodes at time *t*, and $\mathcal {E}^{(t)}$ is the set of hyperedges at time *t*. Each hyperedge is a subset of nodes at time *t* (i.e., $e_i^{(t)} \subseteq \mathcal {V}^{(t)}$). This construction allows the temporal hypergraph to capture the evolution of both node and hyperedge sets over time. We construct our temporal hypergraph using biomedical research papers from the PubMed dataset, which is widely used in HG [3, 4, 8–10]. Each paper is automatically annotated with biomedical concepts (e.g., genes, proteins, diseases, species, chemicals) using PubTator3 [32]. For simplicity, we now refer to the nodes as concepts. We define each paper’s unique set of concepts as a hyperedge $e_i^{(t)}$, where *t* denotes the publication year (though *t* can be any time granularity) and $\mathcal {V}^{(t)}$ is the set of all concepts extracted in that year. For each year *t*, we create a hypergraph $\mathcal {H}^{(t)}$ with hyperedges representing that year’s papers. By sequentially combining these yearly hypergraphs, we obtain the temporal hypergraph $\mathcal {H} = \{\mathcal {H}^{(1)}, \mathcal {H}^{(2)}, \dots , \mathcal {H}^{(T)}\}$, which captures the evolution of biomedical research over time.

### Hard negative sampling

Distinguishing real (positive) hyperedges from fake (negative) ones is critical: false positives waste resources, while false negatives may hide potential breakthroughs. To simulate this challenge, we adopt a hard negative sampling strategy that generates negative hyperedges closely resembling their positive counterparts. Specifically, given a real hyperedge $e_i^{(t)}$, we generate a corresponding negative hyperedge $e_{i,-}^{(t)}$ by randomly replacing a small fraction $r \in [0,1]$ (i.e., the replacement rate) of its concepts. This procedure produces negatives that retain high similarity to the original hyperedge, thereby making it more difficult for the model $f_\theta$, parameterized by $\theta$, to distinguish between the two.

#### Claim 1

Let $e_i^{(t)} \subseteq \mathcal {V}^{(t)}$ be a real hyperedge, and let $e_{i,-}^{(t)}$ be a negative hyperedge obtained by replacing a fraction *r* of its concepts. Then, the ability of the model $f_\theta$ to differentiate $e_i^{(t)}$ from $e_{i,-}^{(t)}$ deteriorates as *r* decreases. In other words, a lower replacement rate results in a negative hyperedge that is more similar to the original, making classification more challenging.

We provide empirical evidence supporting our claim in the Appendix. Based on this evidence, for each real hyperedge $e_i^{(t)}$ in the temporal hypergraph, we generate a hard negative sample by replacing $r = 0.2$ (i.e., 20%) of its concepts. Further, experiments with a method that adversarially learns hard negative samples show that our method learning with our negative sampling strategy leads to better performance.

### Generating trails of ideas

**Algorithm 1:**
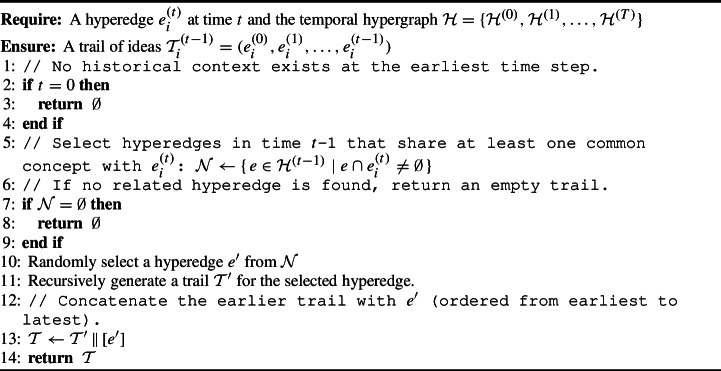
Generating a Trail of Ideas

In the HG setting, new ideas evolve as extensions or modifications of prior ones. To approximate this inheritance, we generate trails of ideas via a temporally constrained random walk over concept-overlapping hyperedges: starting from a target hyperedge, we iteratively select a predecessor that shares at least one concept, thereby simulating how hypotheses build upon past concepts. This selection mirrors the preferential attachment principle [33]; hyperedges with greater concept overlap are more likely to be revisited, thus recovering the rich-get-richer dynamics of idea evolution, and allowing the random walks to recover plausible evolutionary sequences leading to new hypotheses. We call the resulting sequence as a trail of ideas. Starting from a given hyperedge $e_i^{(t)}$ at time $t$, we simulate a trail of ideas by identifying a neighboring hyperedge $e_i^{(t-1)}$ from the previous time step that shares at least one common concept with $e_i^{(t)}$. This process is recursively applied until the earliest time step is reached, thereby forming a trail $\mathcal {T}_i^{(t-1)} = (e_i^{(0)}, e_i^{(1)}, \ldots , e_i^{(t-1)}).$ Algorithm 1 outlines this process. The resulting trail of ideas provides a historic context that approximates the evolution of related hyperedges leading up to $e_i^{(t)}$.

### Temporal hyperedge prediction objective

Our goal is to learn a model $f_\theta$ that, given a hyperedge $e_i^{(t)}$ and its trail of ideas $\mathcal {T}_i^{(t-1)}$, assigns a high probability to $e_i^{(t)}$ being real and a low probability to its corresponding hard negative $e_{i,-}^{(t)}$. Formally, let $\mathbb {P}(e_i^{(t)} \mid \mathcal {T}_i^{(t-1)})$ denote the probability that $e_i^{(t)}$ is true, conditioned on its trail. We formalize our objective as finding parameters $\theta$ that maximize the difference between the log-probability of the true hyperedge and that of the hard negative:

1 $$\begin{aligned} {\theta ^* = \underset{\theta }{\arg \max }\; \; \Biggl [ \log \mathbb {P}\Bigl (e_i^{(t)} \mid \mathcal {T}_i^{(t-1)}\Bigr ) - \log \mathbb {P}\Bigl (e_{i,-}^{(t)} \mid \mathcal {T}_i^{(t-1)}\Bigr ) \Biggr ].} \end{aligned}$$

To achieve this objective, we design $f_\theta$ to integrate temporal, structural, and contextual information from the temporal hypergraph and propose a novel temporal learning framework that leverages both binary cross-entropy and time-anchored contrastive losses to encourage the model to learn evolutionary patterns of hyperedges so that it can effectively distinguish between positive hyperedges and deceptively similar hard negative samples.

### Temporal hypergraph transformer

To capture the temporal evolution of the hypergraph, we design $f_\theta$ to incorporate both its structure and the dynamic evolution of hyperedges. We accomplish this by adopting a transformer-based architecture due to its success in modeling sequences of data across a variety of modalities [34, 35], and success in temporal learning on pairwise graphs [3, 36, 37]. In this section, we explain how our transformer tokenizes a hyperedge with its trail of ideas and generates contextualized embeddings and prediction scores for each hyperedge in the trail.

#### Hyperedge tokenization

A key component of our transformer-based architecture is the conversion of hyperedges into embeddings that capture their structural and temporal properties. Our tokenization process is designed to encode three critical aspects of each hyperedge: its local structure (how it differs from other hyperedges in the same trail), its global structure (its position within the overall hypergraph), and its temporal context (what time the hyperedge belongs to). This rich representation is essential for the transformer to accurately predict future hyperedges.

*Local Awareness.* Local structure distinguishes one hyperedge from another within a trail. To capture the distinguishing structural details, we utilize a HCN to perform convolutions over the hyperedges. A HCN produces embeddings that highlight the unique features of each hyperedge relative to the other hyperedges in the trail [13, 14]. Note that since a HCN is applied on a hyperedge and its trail of ideas, the resulting embeddings are locally contextualized to those hyperedges.

*Global Awareness.* Beyond the local context, the model must understand each hyperedge’s position within the overall hypergraph. To capture this global structure, we incorporate random-walk-based positional encodings. Specifically, we treat the concept features as learnable embeddings, which are initially set using positional encodings derived from random walks [38] on the hypergraph’s bipartite expansion. The process involves converting our hypergraph into a bipartite graph and then computing *L* random-walk transition probabilities using the bipartite graph.

We begin by forming the incidence matrix $\textbf{H} \in \{0,1\}^{N \times M}$, where $\textbf{H}_{j,i} = 1$ if concept *j* belongs to hyperedge *i*, and 0 otherwise. We then construct a bipartite adjacency matrix $\textbf{B} \in \mathbb {R}^{(N+M)\times (N+M)}$, whose two disjoint vertex sets correspond to concepts $\{1,\dots ,N\}$ and hyperedges $\{N+1,\dots ,N+M\}$. The off-diagonal blocks of $\textbf{B}$ are populated with $\textbf{H}$, while its diagonal blocks are set to zero:

$$ \textbf{B} \;=\; \begin{bmatrix} \textbf{0}_{N \times N} &  \textbf{H} \\ \textbf{H}^\top &  \textbf{0}_{M \times M} \end{bmatrix}. $$

Each row of $\textbf{B}$ is then normalized by its degree (the sum of all the entries in that row) so that every row adds up to 1. This process ensures that, from any row (concept or hyperedge), the distribution of edge weights naturally forms a probability distribution across its neighbors.

Let $\textbf{B}^1 = \textbf{B}$. For $l = 2, \dots , L$, let $\textbf{B}^l = \textbf{B}^{l-1} \textbf{B}$. This repeated multiplication captures *l*-step random-walk transition probabilities. For each concept $j \in \{1,\dots ,N\}$, we collect the diagonal entry $(\textbf{B}^l)_{j,j}$ for each $l = 1, \dots , L$. This value encodes the probability that a random walk starting at *j* returns to *j* in *l* steps. By stacking these diagonal entries across all *l*, we obtain a *L*-dimensional embedding for each concept. Finally, we assemble these embeddings into the positional encoding matrix $\textbf{PE} \in \mathbb {R}^{N \times L}$, where $\textbf{PE}_{j,:}$ represents the random-walk embedding of node *j*. In our implementation, we set the walk length $L=5$. We chose $L=5$ based on the empirical observation that random-walk length exhibits a trade-off in graph learning: while longer walks provide broader coverage of the graph structure, performance improvements plateau as walks become sufficiently long, exhibiting diminishing returns [39]. This choice balances capturing multi-hop neighborhood structure with computational efficiency. We pad the positional encodings of the concepts with zeros so that they become *d*-dimensional.

The positional encodings capture multi-hop connectivity patterns, encoding long-range topological relationships and providing each hyperedge with properties of its position in the entire graph. Making the concept features learnable also allows hyperedge embeddings to reflect both the static global structure and evolving multi-concept interactions across the entire hypergraph. Details on how these positional encodings are derived are documented in our codebase.

*Temporal Encoding.* Temporal context is vital for modeling the evolution of hyperedges over time. Based on the components described above, each hyperedge is first encoded in a vector $\textbf{z}_i^{(t)}$ that captures local and global awareness of the trail and hypergraph. To integrate temporal information, we combine hyperedge embeddings with time encodings [37, 40], which are vector representations that capture the temporal properties of each time step. Each hyperedge embedding is concatenated with a learnable time encoding $\psi (t)$ specific to the time step $t$, where $\psi (t) = \text {cosine}(Wt+b)$ is the time encoding function and $W \in \mathbb {R}^{d_{\text {time}}}$ and $b \in \mathbb {R}$ are learnable parameters and $d_{\text {time}}$ is the dimension of the time encoding $\textbf{z}_i^{(t)} \leftarrow \text {Concat}(\textbf{z}_i^{(t)}, \psi (t)).$ Here, $\text {Concat}$ concatenates the embedding with its time encoding so that $\textbf{z}_i^{(t)}$ becomes a vector in $\mathbb {R}^{d_{\text {token}}}$, where $d_{\text {token}} = d + d_{\text {time}}$. This embedding serves as the tokenized hyperedge, enriched with local and global structural information as well as temporal context.

#### Time-aware masked hyperedge attention

To capture temporal dependencies in hyperedge evolution, we introduce Time-aware Masked Hyperedge Attention, a masked attention mechanism that produces contextualized embeddings for each hyperedge such that every embedding encapsulates both the intrinsic properties of that hyperedge and the historical context provided by all prior hyperedges in the trail. By ensuring that each hyperedge only attends to itself and hyperedges occurring before it, this mechanism preserves the temporal order and accurately models the evolution of ideas. Although a trail of ideas is defined as $\mathcal {T}_i^{(t-1)} = (e_i^{(0)}, e_i^{(1)},\dots , e_i^{(t-1)})$, our transformer input also includes the current hyperedge we wish to evaluate $e_i^{(t)}$, which are tokenized to form a matrix $\textbf{Z}_i = [\textbf{z}_i^{(0)}, \textbf{z}_i^{(1)}, \ldots , \textbf{z}_i^{(t)}]$. With $\textbf{Z}_i$, we compute the query, key, and value matrices as:

2 $$\begin{aligned} \textbf{Q} = \textbf{Z}_i \textbf{W}_Q, \quad \textbf{K} = \textbf{Z}_i \textbf{W}_K, \quad \textbf{V} = \textbf{Z}_i \textbf{W}_V, \end{aligned}$$

with learnable projection matrices $\textbf{W}_Q, \textbf{W}_K, \textbf{W}_V \in \mathbb {R}^{d_\text {token} \times d_\text {token}}$. This step transforms the tokenized hyperedge representations into vectors that can interact via the attention mechanism, setting the stage for incorporating historical context.

To ensure that each hyperedge’s embedding only incorporates information from itself and its preceding hyperedges (thereby preventing leakage of future information), we apply a masked scaled dot-product attention, referred to as $\text {MaskedAttention}$:

3 $$\begin{aligned} {\text {MaskedAttention}(\textbf{Q}, \textbf{K}, \textbf{V}) = \text {softmax} \left( \frac{\textbf{Q} \textbf{K}^\top + M}{\sqrt{d_{\text {token}}}} \right) \textbf{V},} \end{aligned}$$

where the mask *M* prevents any hyperedge from attending to future time steps [34].

To capture multiple patterns of temporal dependencies, we adopt a multi-head attention approach. Here, the masked attention operation is performed in parallel across *H* heads, each with its own set of projection matrices. The outputs of these heads are concatenated and linearly transformed:

4 $$\begin{aligned} {\text {MultiHead}(\textbf{Q}, \textbf{K}, \textbf{V}) = \text {Concat} \left( \text {head}_1, \dots , \text {head}_H \right) \textbf{W}_O,} \end{aligned}$$

where each head independently learns attention weights, $\text {Concat}$ concatenates the heads together and $\textbf{W}_O$ projects the concatenated outputs back to a $d_{token}$-dimensional space. Following [34], the output of the multi-head attention is added to a residual connection from the original tokenized hyperedges and then normalized using layer normalization. This is further processed through a feed-forward network, ultimately producing the final contextualized embeddings $[\textbf{h}_i^{(0)}, \textbf{h}_i^{(1)}, \ldots , \textbf{h}_i^{(t)}]$. They are then used to generate prediction scores for each of the hyperedges in the trail via a sigmoid-gated multi-layer perceptron ($\text {MLP}$):

5 $$\begin{aligned} [s_i^{(0)}, s_i^{(1)}, \ldots , s_i^{(t)}] = \text {MLP}([\textbf{h}_i^{(0)}, \textbf{h}_i^{(1)}, \ldots , \textbf{h}_i^{(t)}]). \end{aligned}$$

The design of Time-aware Masked Hyperedge Attention enriches each hyperedge embedding with both its intrinsic features and the historical context from preceding hyperedges, enabling the model to accurately capture temporal dynamics and generate reliable predictions.

**Algorithm 2:**
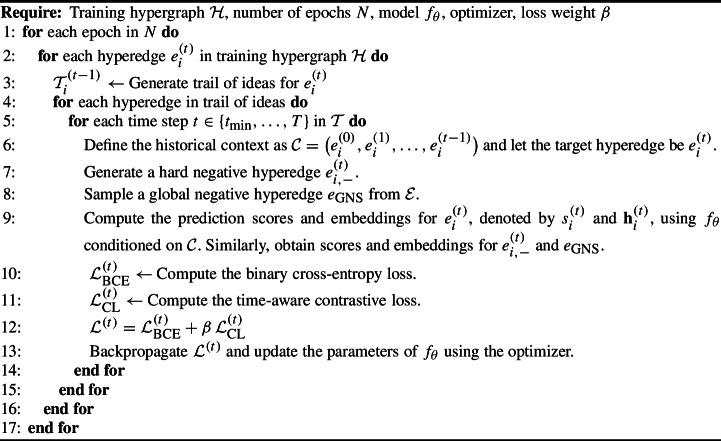
Training Algorithm

### Temporal hypergraph contrastive learning

We introduce our novel training framework that optimizes the objective described in Eq. 1. Our approach integrates binary cross-entropy loss with global negative sampling and a time-anchored contrastive loss to enforce evolutionary consistency in the model’s embeddings. By anchoring each prediction in the historical context of prior hyperedges, our framework compels the model to distinguish true hyperedges from hard negatives, even when the negatives are deceptively similar, thereby capturing the natural progression of multi-concept interactions in the temporal hypergraph.

*Binary Classification Objective with Global Negative Sampling.* To ensure our model generalizes to truly unseen hyperedges from future timesteps, we incorporate global negative sampling. In addition to locally generated hard negatives we also sample a global negative hyperedge drawn randomly from the entire hypergraph (excluding $$e_i^{(t)}$$). Global negative samples represent entirely unrelated hypotheses which can be from any time step, forcing the model to distinguish plausible evolutionary continuations from arbitrary, out-of-context hyperedges. This helps the model generalize to unseen timestamps. The prediction scores generated for the positive hyperedge ($$s_i^{(t)}$$), its hard negative ($$ s_{i,-}^{(t)}$$), and the global negative sample ($$s_{\text {GNS}}$$) are incorporated into training with the following binary-cross-entropy loss:

6 $$\begin{aligned} { \mathcal {L}_{\text {BCE}, i}^{(t)} = - \log \bigl (s_i^{(t)}\bigr ) - \text {log}(1 - s_{i,-}^{(t)}) - \text {log}(1 - s_{\text {GNS}}).} \end{aligned}$$

*Time-anchored Contrastive Loss.* Claim 1 demonstrates that as the replacement rate *r* decreases, negative hyperedges become increasingly similar to their real counterparts, making discrimination more challenging. To overcome this, our training objective incorporates a margin-based contrastive loss [41] that enforces a separation margin between the historical context and the embeddings of both positive and negative hyperedges. In this formulation, $\textbf{h}_i^{(t-1)}$ denotes the historical context embedding summarizing the evolution up to time $t-1$, $\textbf{h}_i^{(t)}$ is the embedding of the positive hyperedge at time *t*, $\textbf{h}_{i,-}^{(t)}$ is the embedding of a locally generated negative hyperedge, and $\textbf{h}_{\text {GNS}}$ is the embedding for the global negative hyperedge. We define the time-anchored contrastive loss as:

7 $$\begin{aligned} \begin{aligned} \mathcal {L}_{\text {CL},i}^{(t)} =\;2\alpha \,\Vert \textbf{h}_i^{(t-1)}-\textbf{h}_i^{(t)}\Vert ^2+(1-\alpha )\Bigl [\max \bigl (0,\textrm{Margin}-\Vert \textbf{h}_i^{(t-1)}-\textbf{h}_{i,-}^{(t)}\Vert _2\bigr )^2\\ \quad +\max \bigl (0,\textrm{Margin}-\Vert \textbf{h}_i^{(t-1)}-\textbf{h}_{\textrm{GNS}}\Vert _2\bigr )^2\Bigr ]. \end{aligned} \end{aligned}$$

where the first term pulls the positive hyperedge embedding closer to the historical context, and the remaining terms push both the local and global negative embeddings away from it. The hyperparameters $\alpha \in [0,1]$ balances these objectives, and $\text {Margin}$ specifies the minimum desired separation.

*Implication of time-anchored contrastive loss.* In a setting where it is difficult for a model to distinguish between a positive and hard negative hyperedge, leveraging the temporal evolution captured by the historical embedding $\textbf{h}_i^{(t-1)}$ becomes critical. This motivates us to make the following claim:

#### Claim 2

Let $e_i^{(t)}$ be a real hyperedge at time t with its contextualized embedding $\textbf{h}_i^{(t)}$, and let $e_{i,-}^{(t)}$ be a hard negative sample derived from $e_i^{(t)}$ with embedding $\textbf{h}_{i,-}^{(t)}$. Suppose that the temporal hypergraph transformer model $f_\theta$ trained with the time-anchored contrastive loss integrates historical context via an embedding $\textbf{h}_i^{(t-1)}$ that summarizes the progression of hyperedges from previous time steps, and assume that positive hyperedges evolve in a predictable manner so that the current true hyperedge (at time *t*) is expected to be close in embedding space to the historical context. Then, even when hard negative samples are generated with a low replacement rate *r*, we have:

8 $$\begin{aligned} \Vert \textbf{h}_i^{(t-1)} - \textbf{h}_i^{(t)}\Vert _2 < \Vert \textbf{h}_i^{(t-1)} - \textbf{h}_{i,-}^{(t)}\Vert _2. \end{aligned}$$

In other words, even when a hard negative is generated, the inclusion of temporal context and time-anchored contrastive loss helps the positive hyperedge embedding remain closer to the historical context than its negative counterpart.

Claim 2 shows that by anchoring predictions in historical context, the time-anchored contrastive loss both separates true hyperedge embeddings from their negatives and implicitly regularizes the temporal consistency of the learned representations. This is grounded in a time-adapted distributional hypothesis [42], which suggests that if knowledge evolves gradually, then hyperedges at time $t$ should be semantically close to those at $t-1$; our contrastive loss explicitly enforces this inductive bias, encouraging such consistency. As a result, the model captures meaningful evolutionary patterns, improving generalization to future time steps and yielding a latent space that mirrors the natural progression of biomedical advancements. In the Appendix we report and discuss empirical results which show that time-anchored contrastive loss does result in the real hyperedge embeddings $\textbf{h}_i^{(t)}$ being closer to their historical context $\textbf{h}_i^{(t-1)}$ than the negative embeddings $\textbf{h}_{i,-}^{(t)}$.

*Overall Training Objective.* The overall loss over the entire hypergraph is:

9 $$\begin{aligned} \mathcal {L} = \frac{1}{|\mathcal {E}|} \sum _{i \in \mathcal {E}} \sum _{k=0}^t \Bigl ( \mathcal {L}_{\text {BCE},i}^{(k)} + \beta \, \mathcal {L}_{\text {CL},i}^{(k)} \Bigr ). \end{aligned}$$

Here, the outer summation accounts for all the hyperedges in the hypergraph. The inner summation accounts for each hyperedge currently being evaluated as well as its trail of ideas. $\beta \in [0,1]$ controls the contribution of the contrastive loss relative to the BCE loss. Algorithm 2 outlines how HyHG trains with temporal hyperedge contrastive learning. Time-complexity details are provided in the Appendix.

**Table 1:** The number of concepts and hyperedges per split

	Neurology	Immunology	Embryology
Num. Concepts	20,563	72,986	133,629
Train (2000–20)	11,052	13,809	41,399
Val. (2021–22)	3,447	4,900	6,786
Test (2023–24)	1,550	2,390	3,097

**Table 2:** Experimental results on the Neurology, Immunology, and Embryology datasets. Rows are grouped into four categories: pairwise graph methods, hypergraph methods, temporal hypergraph methods, and our method (HyHG). The CV column shows consistency (coefficient of variation) across datasets based on the AUC metric. Bold numbers indicate the best overall scores, while underlined numbers show the best results within each group

**Method**	**CV (%)**	*Neurology*					*Immunology*					*Embryology*				
		AUC	Acc	Prec	F1	Avg. Prec	AUC	Acc	Prec	F1	Avg. Prec	AUC	Acc	Prec	F1	Avg. Prec
GCN	6.3%	0.526	0.504	0.502	0.661	0.538	0.523	0.501	0.501	0.663	0.553	0.584	0.498	0.499	0.661	0.590
GAT	9.0%	0.550	0.543	0.557	0.478	0.553	0.616	0.500	0.500	0.666	0.577	0.517	0.500	0.500	0.667	0.535
GraphTransformer	4.5%	0.578	0.559	0.568	0.527	0.551	0.534	0.540	0.552	0.479	0.525	0.537	0.538	0.561	0.429	0.521
TGAT	5.3%	0.606	0.560	0.599	0.452	0.584	0.608	0.546	0.559	0.490	0.569	0.664	0.628	0.631	0.624	0.626
AHP	61.2%	0.764	0.500	0.500	0.667	0.735	0.844	0.802	0.716	0.834	0.791	0.177	0.500	0.500	0.667	0.339
CACHE	4.7%	0.735	0.662	0.676	0.655	0.710	0.698	0.560	0.593	0.517	0.720	0.670	0.577	0.595	0.556	0.687
UniGCN	2.7%	0.607	0.548	0.560	0.497	0.563	0.603	0.541	0.553	0.478	0.560	0.634	0.537	0.560	0.425	0.579
UniGAT	6.5%	0.600	0.546	0.560	0.491	0.560	0.605	0.541	0.554	0.479	0.561	0.537	0.536	0.560	0.423	0.522
CHESHIRE	3.4%	0.620	0.576	0.611	0.497	0.607	0.659	0.629	0.635	0.621	0.620	0.623	0.584	0.610	0.529	0.606
HypergraphConv	**1.6%**	0.614	0.544	0.557	0.485	0.567	0.611	0.572	0.583	0.542	0.564	0.629	0.615	0.585	0.674	0.578
UniGCN + THT	2.8%	0.725	0.644	0.778	0.530	0.735	0.694	0.637	0.649	0.620	0.701	0.688	0.622	0.597	0.666	0.686
UniGAT + THT	2.6%	0.715	0.575	0.544	0.685	0.720	0.709	0.619	0.580	0.693	0.709	0.681	0.628	0.603	0.668	0.665
CHESHIRE + THT	5.8%	0.744	0.675	0.745	0.621	0.734	0.762	0.679	0.877	0.564	0.800	0.681	0.642	0.642	0.643	0.736
HypergraphConv + THT	2.9%	0.728	0.661	0.664	0.658	0.737	0.719	0.661	0.692	0.631	0.721	0.689	0.648	0.661	0.633	0.676
UniGCN + HyHG	4.6%	0.887	0.813	0.829	0.808	0.884	0.841	0.755	0.738	0.764	0.852	0.809	0.744	0.722	0.756	0.836
UniGAT + HyHG	5.8%	0.929	0.877	0.848	0.882	0.930	0.840	0.760	0.739	0.771	0.849	0.843	0.797	0.793	0.798	0.866
CHESHIRE + HyHG	5.2%	0.776	0.735	0.761	0.720	0.790	0.859	0.781	0.777	0.783	0.884	0.801	0.742	0.734	0.745	0.842
**HypergraphConv + HyHG**	4.4%	**0.959**	**0.918**	**0.889**	**0.921**	**0.958**	**0.957**	**0.922**	**0.899**	**0.924**	**0.956**	**0.887**	**0.838**	**0.819**	**0.843**	**0.904**

## Experiments

In this section we present our experiment results. Our goal is to demonstrate the efficacy of our method and show the contribution of each of our framework’s components. We also provide case studies that illustrate how our framework learns the temporal evolution of hyperedges to rediscover biomedical advancements.

### Experimental setup

Since validating novel hypotheses often requires costly wet-lab experiments, we follow standard practice and evaluate retrospectively by training on historical literature and testing on future, held-out publications [3, 4, 8–10, 43]. We construct three temporal hypergraph datasets from PubMed articles in Neurology, Immunology, and Embryology, partitioned into training (2000–2020), validation (2021–2022), and testing (2023–2024) sets based on publication year. We adjust the granularity of our timesteps to the annual level to match the yearly publication patterns, similar to [43], although our method can support any time granularity. To prevent data leakage, we remove all hyperedges in the validation and test sets that have equivalent hyperedges in the training set. Additionally, when evaluating our method, we randomly split the validation set in half, using only one half for model optimization so that the other half remains available for inclusion in the test-time trail of ideas. Table 1 summarizes dataset splits and statistics. We compare our method against state-of-the-art pairwise models (GCN [44], GAT [45], GraphTransformer [46], TGAT [37]), HGNNs (AHP [18], CACHE [16]), HCNs (HypergraphConv [12], CHESHIRE [14], UniGAT [13], UniGCN [13]), and the Temporal Hypergraph Transformer (THT), which processes hyperedges in batches without a trail-of-idea generation or global awareness. For each positive hyperedge, one hard negative sample is generated. Performance is evaluated using AUC, Accuracy (Acc.), Precision (Prec.), F1 Score (F1), and Average Precision (Avg. Prec.) metrics. All models are trained on the training hypergraph and evaluated on the test set. Additional details about the baselines and training are in the Appendix and more data and implementation details are documented in the codebase.

**Table 3:** Ablation study results across Neurology, Immunology, and Embryology datasets. The study ablates Global Awareness (GA), global negative sampling (GNS), and Time-anchored Contrastive Learning (TACL)

**Method**	*Neurology*					*Immunology*					*Embryology*				
	AUC	Acc	Prec	F1	Avg. Prec	AUC	Acc	Prec	F1	Avg. Prec	AUC	Acc	Prec	F1	Avg. Prec
HyHG w/o GA	0.744	0.714	0.658	0.757	0.760	0.898	0.846	0.852	0.845	0.898	0.858	0.777	0.756	0.785	0.879
HyHG w/o GNS	0.825	0.736	0.686	0.767	0.818	0.893	0.854	0.855	0.854	0.891	0.829	0.751	0.737	0.758	0.857
HyHG w/o TACL	0.799	0.706	0.643	0.758	0.802	0.860	0.765	0.710	0.792	0.864	0.854	0.785	0.765	0.793	0.877
HyHG	**0**.**959**	**0**.**918**	**0**.**889**	**0**.**921**	**0**.**958**	**0**.**957**	**0**.**922**	**0**.**899**	**0**.**924**	**0**.**956**	**0**.**887**	**0**.**838**	**0**.**819**	**0**.**843**	**0**.**904**

**Table 4:** Case study of HyHG rediscovering an Immunology advancement. The table presents the rediscovered study (left column) alongside three influential context studies that trace the emergence relevant concepts over time

Rediscovered Study	Context Study 1	Context Study 2	Context Study 3
**PMID:**37496680, **Year:**2023 **Concepts:****Description:** This rediscovered study uncovers a novel mechanism where neutrophils use *NMDA* receptor-mediated calcium signaling and glutamate release to facilitate intercellular communication, potentially influencing immune responses and inflammation	**PMID:**21248269, **Year:**2011 **Overlapping Concepts:****Relevance:** The concepts *Sodium Chloride, Potassium Chloride*, and Magnesium Chloride were extracted from a 2011 paper. These concepts introduce important chemicals relevant to the rediscovery’s context. Each of these chemicals relate to the methods and experiment used to facilitate the rediscovery	**PMID:**30200565, **Year:**2018 **Overlapping Concepts:****Relevance:** The concepts *Immunologic Deficiency Syndromes, NAD, Infections*, and *Aldehydes* are extracted from a 2018 study. *NAD* and *Aldehydes* are relevant to the experimental techniques used in the rediscovery, while *Immunologic Deficiency Syndromes* and *Infections* are concepts that relate to the rediscovery’s domain	**PMID:**35480624, **Year:**2022 **Overlapping Concepts:****Relevance:** These concepts come from a 2022 study. *Inflammation* supports the rediscovery’s focus on neutrophil-driven inflammation. *Oxalobacter formigenes* and *Infections* align with neutrophil involvement in microbial defenses, while *Nitrogen* and *Carbon Dioxide* relate to roles for environmental factors in cellular signaling

**Table 5:** Case study of HyHG rediscovering an Neurology advancement. The table presents the rediscovered study (left column) alongside three influential context studies that trace the emergence relevant concepts over time

Rediscovered Study	Context Study 1	Context Study 2	Context Study 3
**PMID:**37055214, **Year:**2023 **Concepts:****Description:** This rediscovered study concluded that the "Hand-Arm Bimanual Intensive Therapy Including Lower Extremities" protocol is a feasible, intensive rehabilitation approach for improving motor function, coordination, and quality of life in adults with chronic stroke, supported by comprehensive motor, cognitive, and neuroimaging assessments	**PMID:**11594918, **Year:**2001 **Overlapping Concepts:****Relevance:** The concepts *Mental Disorders, Cognition Disorders, ADHD*, and *Multiple Sclerosis* were extracted from a 2001 article. These concepts appear in the 2023 rediscovery and relate to strokes and conditions linked with strokes	**PMID:**24002980, **Year:**2013 **Overlapping Concepts:****Relevance:** The concepts *Stroke, Cerebral Hemorrhage, Human*, and *Mental Disorders* appear together in a 2013 study and a overlap of the rediscovery’s concepts. This context includes more concepts closely related to the strokes and conditions related of strokes for humans	**PMID:**25024566, **Year:**2014 **Overlapping Concepts:****Relevance:** In addition to *Mental Disorders* and *Stroke,* (introduced in previous contexts) a 2014 study introduces the overlapping concepts of *Aphasia*, a common consequence of strokes involving language and communication impairments, and *Paresis*, a sort of partial paralysis often addressed in stroke rehabilitation

**Table 6:** Case study of HyHG rediscovering an Embryology advancement. The table presents the rediscovered study (left column) alongside three influential context studies that trace the emergence relevant concepts over time

Rediscovered Study	Context Study 1	Context Study 2	Context Study 3
**PMID:**37382283,**Year:**2024 **Concepts:****Description:** This rediscovered study found that higher paternal body mass index (BMI) negatively affects fertilization rates, embryo quality, and increases neonatal risks such as macrosomia, emphasizing the impact of paternal health on assisted reproductive technology (ART) outcomes	**PMID:**36361577, **Year:**2022 **Overlapping Concepts:****Relevance:** These overlapping concepts are introduced to the rediscovery’s context when extracted together from a 2022 study. *CGB5* is a gene essential to the ‘in vitro fertilization’ process described in the rediscovery. *Spontaneous Abortion* and *Inflammation* are consequences to a high BMI and affect reproductivity	**PMID:**29675488, **Year:**2018 **Overlapping Concepts:****Relevance:** These overlapping concepts are introduced to the rediscovery’s context when extracted together from a 2018 study. *Lipids* relate to the relate to the rediscovery’s discussion on BMI and sperm health, while *Infertility* and *Ovarian Diseases* contribute to the overall discussion on reproduction in the rediscovery	**PMID:**23877792, **Year:**2013 **Overlapping Concepts:****Relevance:** The concepts *Estradiol, Glucose*, and *Embryo Loss* are introduced to the rediscovery’s context together from a 2013 study. *Estradiol* and *Glucose* are two factors related to increases BMI discussed in the rediscovered study. *Embryo Loss* is a reproductive outcome relevant to the rediscovery

### Results

We report our main results in Table 2 and find the following insights: (1) Modeling publications as hyperedges that capture complex, multi-concept interactions leads to superior performance over pairwise methods. Table 2 shows that traditional pairwise models consistently underperform compared to hypergraph-based methods, which better capture higher-order relationships. (2) Extending hypergraph models with temporal modeling results in performance gains. Incorporating a THT improves AUC across all HCN baselines. For instance, HypergraphConv achieves an AUC of 0.611 on the Immunology dataset, whereas its temporal extension (HypergraphConv + THT) raises it to 0.719, demonstrating the value of modeling temporal dependencies. (3) Our full method achieves the highest performance across all datasets by integrating trail-of-idea generation and time-anchored contrastive learning. HypergraphConv with HyHG achieves AUC scores of 0.959, 0.957, and 0.887 on the Neurology, Immunology, and Embryology datasets, respectively, outperforming all baselines. This result highlights how explicitly leveraging temporal evolution and contrastive training refines predictive power. (4) HyHG seamlessly adapts static HCNs to the temporal domain, consistently outperforming their static counterparts. The performance gap between HypergraphConv and HypergraphConv + HyHG (AUC: 0.614 → 0.959 in Neurology) underscores how modeling temporal context significantly enhances hypergraph-based hypothesis generation.

### Ablation study

Our ablation study (Table 3) quantifies the contributions of key components in HyHG. Removing global awareness leads to notable performance drops, confirming that modeling long-range dependencies is crucial for robust hyperedge representations. For example, in Neurology, AUC drops from 0.959 to 0.744, highlighting the role of positional encoding in preserving structural context. Eliminating global negative sampling reduces the model’s ability to differentiate plausible from arbitrary hypothesis continuations, leading to lower discriminative power. Across all datasets, we observe a decrease in all metrics (e.g., AUC 0.957 → 0.893 in Immunology), demonstrating that leveraging temporally challenging negatives strengthens contrastive learning. The time-anchored contrastive loss plays a critical role in enforcing temporal consistency, as its removal causes a large performance drop among all datasets and metrics, illustrating that contrastive constraints help separate plausible hypotheses from deceptively similar ones.

To quantify the relative importance of each component, we compute their absolute and relative contributions to HyHG’s overall performance. Using Neurology as a representative example, Global Awareness (GA) contributes 0.215 AUC points (22.4% of full performance), accounting for 42.2% of the total performance loss when removed. Time-anchored Contrastive Loss (TACL) contributes 0.160 AUC points (16.7%), representing 31.4% of total loss. Global Negative Sampling (GNS) contributes 0.134 AUC points (14.0%), accounting for 26.3% of total loss. Across Immunology and Embryology, the pattern remains consistent: GA is most critical for Neurology (22.4% loss) but less so for Embryology (3.3% loss), while TACL maintains critical importance across Immunology (10.1% loss). This domain-specific variation suggests that Neurology’s concept structure benefits most from global structural awareness via random walks, while temporal consistency (TACL) is universally important. Overall, GA and TACL are roughly equally important in driving HyHG’s superior performance over temporal baselines.

### Dataset difficulty and method progression

To assess relative dataset difficulty and validate the importance of temporal modeling, we analyzed performance progression across method categories. Embryology is the most challenging dataset, with static baseline methods achieving 0.545 mean AUC compared to temporal methods’ 0.685 mean AUC on Embryology, reflecting a larger performance gap than other datasets. Across all three datasets, static methods average 0.624 AUC, while temporal methods average 0.711. HyHG variants consistently achieve strong performance across all domains (mean 0.835 AUC, with HypergraphConv + HyHG reaching 0.934), demonstrating robust generalization despite varying dataset difficulty.

The performance progression shows clear improvements with each methodological advancement. Pairwise methods achieve 0.570 mean AUC; static hypergraph methods achieve 0.624 AUC (+9.4% over pairwise); temporal methods achieve 0.711 AUC (+14.0% over static). HyHG with HypergraphConv reaches 0.934 AUC (+31.4% over temporal methods), representing a substantially larger improvement than the temporal-only step. The HyHG improvement is 2.2x larger than the temporal-only improvement, demonstrating that trail-of-ideas generation combined with time-anchored contrastive learning provide significant gains beyond basic temporal modeling. Note that the HypergraphConv variant achieves the strongest performance, while the average across all HyHG variants is 0.866 AUC (+21.7% over temporal). The consistency of HyHG (coefficient of variation: 4.4%) across datasets compared to most baselines further demonstrates robust generalization.

### Method consistency and generalization

We analyzed the consistency of all methods across datasets to evaluate generalization capability by computing the coefficient of variation (CV) across datasets for each method. The CV metrics in Table 2 reveal important insights about method robustness. HyHG (HypergraphConv configuration) achieves a coefficient of variation of 4.4% (mean AUC 0.934, std 0.041), maintaining consistent performance across Neurology (0.959), Immunology (0.957), and Embryology (0.887). While the base architecture HypergraphConv achieves lower CV at 1.6%, the temporal variants of HypergraphConv (HypergraphConv + THT at 2.9% CV) and HyHG (4.4% CV) demonstrate that adding temporal modeling does not significantly compromise consistency. Notably, HyHG achieves the highest mean AUC (0.934) while maintaining moderate CV (4.4%), representing a favorable trade-off between performance and generalization. Compared to other high-performing methods, HyHG shows better consistency than GAT (9.0% CV) and UniGAT + HyHG (5.8% CV), indicating that the contrastive learning approach provides effective generalization despite the complexity of temporal modeling.

### Case studies

We present three case studies in Tables 4, 5, and 6, where HyHG predicts a hyperedge that matches the set of concepts extracted from a future publication, highlighting recent immunology, neurology, and embryology advancements, respectively.

In immunology, our method rediscovered a hyperedge from PMID: 37496680 that captured neutrophil-mediated NMDA receptor signaling, which may influence the inflammatory response. This hyperedge includes concepts such as Inflammation, Immunologic Deficiency Syndromes, NAD, and Infections. Within the rediscovery’s trail of ideas, a 2011 study (PMID: 21248269) provided additional context by introducing Sodium Chloride, Potassium Chloride, and Magnesium Chloride. Later, a 2018 study (PMID: 30200565) contributed the concepts NAD, Aldehydes, and Immunologic Deficiency Syndromes, and by 2022 (PMID: 35480624), microbial interactions were highlighted with Inflammation, Oxalobacter formigenes, and Carbon Dioxide.

In neurology, our framework rediscovered a hyperedge from PMID: 37055214 that encapsulates a key rehabilitation protocol for elderly stroke patients, including concepts like Stroke, Cerebral Hemorrhage, Paresis, and Aphasia. Within the rediscovery’s trail of ideas, a 2001 study (PMID: 11594918) introduced related concepts such as Cognitive Disorders, Multiple Sclerosis, and ADHD. These were expanded in a 2013 study (PMID: 24002980) to include Stroke, Cerebral Hemorrhage, and Mental Disorders, and by 2014 (PMID: 25024566), Aphasia and Paresis were further emphasized, highlighting the cognitive and motor deficits associated with stroke.

In embryology, our framework rediscovers a study (PMID: 37382283) linking paternal BMI to reduced fertility and neonatal risks incorporated *Estradiol, Lipids, Spontaneous Abortion,* and *Infertility*. The 2013 study (PMID 23877792) introduced *Estradiol, Glucose,* and *Embryo Loss*, followed by a 2018 study (PMID 29675488) that expanded on *Lipids, Infertility,* and *Ovarian Diseases*. By 2022 (PMID 36361577), genetic and inflammatory factors, including *CGB* and *Inflammation*, further enriched the rediscovery’s context. These case studies demonstrate how our model effectively predicts and contextualizes biomedical discoveries by integrating historical knowledge from diverse sources.

### Discussion

#### Robustness to noise from PubTator3

Our framework relies on automated concept extraction via PubTator3. While automated extraction inevitably introduces noise, HyHG is designed to be robust against individual entity errors through structural filtering and temporal contrastive learning.

First, to improve interpretability and reduce noise, we discard hyperedges with fewer than 5 or more than 30 concepts. Despite this filtering, HyHG effectively handles varying hyperedge sizes (mean $\approx 11$, std $\approx 6$). Empirical results in Table 2 demonstrate this robustness: HyHG consistently achieves state-of-the-art performance (e.g., AUC > 0.95 in Neurology and Immunology) across diverse datasets, indicating that the model is not sensitive to domain-specific noise patterns.

Second, our time-anchored contrastive learning objective emphasizes temporal consistency over isolated terms. By modeling multi-concept hyperedges, the model leverages broader context, reducing sensitivity to individual concept misidentification. The critical role of this mechanism is confirmed by our ablation study (Table 3); removing the time-anchored contrastive loss (TACL) results in significant performance drops across all datasets. This confirms that the contrastive objective is the primary driver of the model’s resilience.

Third, HyHG outperforms AHP (Table 2), which uses adversarial training to generate hard negatives. We hypothesize that temporal context is the primary driver of discrimination: by anchoring predictions in historical hyperedge sequences, the time-anchored contrastive loss provides sufficient discriminative signal, making complex adversarial negative generation unnecessary. Our simpler strategy of replacing *r* concepts in real hyperedges, combined with temporal anchoring, thus achieves stronger performance.

#### Predicting novel research ideas

A common challenge in evaluating Hypothesis Generation (HG) is the reliance on binary classification as a proxy for generative capability, however, HyHG is not merely learning a static decision boundary; it models the emergence of scientific ideas.

Valid predictions made by HyHG are aligned with the learned trajectory of scientific discovery. This is quantitatively supported by the high precision and AUC scores on the held-out 2023-2024 test sets in Table 2, representing successful "rediscovery" of future findings. Qualitatively, this capability is illustrated in our case studies (Tables 4, 5, and 6), where HyHG correctly predicted complex, multi-concept advancements. For instance, in the Immunology dataset (Table 4), the model successfully identified the novel association between neutrophils and NMDA receptor signaling by integrating historical trails involving sodium chloride and varying immunologic syndromes. This evidence suggests that HyHG captures "paradigm-shifting" discoveries—novel concept combinations that align with latent evolutionary patterns—rather than merely memorizing incremental trends.

Importantly, our evaluation setup ensures that test hyperedges are previously unseen: we remove all hyperedges in the validation and test sets that have equivalent hyperedges in the training set (see § 4). Thus, the model predicts hyperedges whose exact concept combinations did not appear in training, even when individual concepts may have. This supports the claim that HyHG generates novel hypotheses rather than simply recalling past patterns.

## Conclusion

We introduced HyHG, a temporal hypergraph contrastive learning framework for hypothesis generation that models multi-concept interactions and their evolution. By generating idea trails, contextualized hyperedge embeddings, and enforcing temporal patterns through time-anchored contrastive learning, HyHG outperforms existing methods in future hyperedge prediction, showing its potential to uncover novel biomedical insights.

## Data Availability

We created hypergraphs from the PubMed database. Our datasets can be publicly accessed from GitHub via the following link: https://github.com/amir-hassan25/Temporal-Hypergraph-Contrastive-Learning.

## Additional experimental details

### A.1 baseline details

- **Graph convolution network (GCN) **[44]: The GCN generalizes the convolution operation to graph-structured data by aggregating and transforming features from each concept’s local neighborhood. This layer-wise aggregation scheme allows GCN to effectively capture localized structural information in the pairwise graph.
- **Graph attention network (GAT) ** [45]: The GAT introduces an attention mechanism that assigns varying levels of importance to neighbors when aggregating features. By learning dynamic attention coefficients, it can capture complex relationships among pairwise concept interactions.
- **GraphTransformer** [46]:The GraphTransformer adapts the Transformer architecture to graph data by leveraging self-attention to capture both local and long-range dependencies.
- **CACHE** [16]: CACHE is a hypergraph neural network that represents hypergraphs as bipartite graphs. It uses an self-attention based architecture and has a counterfactual loss function to perform hyperedge prediction in an explainable manner.
- **UniGCN** [13]: UniGCN is a hypergraph convolution network that uses the UniGNN framework to generalize the convolution used in the GCN to the hypergraph setting.
- **UniGAT** [13]: UniGAT is a hypergraph convolution network that uses the UniGNN framework to generalize the convolution used in the GAT to the hypergraph setting.
- **AHP** [18]: AHP adversarial learning to generate negative hyperedges for robust hyperedge prediction.
- **CHESHIRE** [14]: CHESHIRE is a hypergraph convolution network that, when conducting convolutions on hypergraphs, uses a Chebyshev spectral graph convolution network over clique expansion of each hyperedge.
- **HypergraphConv** [12]: HypergraphConv conducts convolutions to hypergraphs by propagating vertex features through hyperedges using a normalized aggregation operator that weights hyperedge importance. It includes symmetric normalization to ensure stable gradient propagation when stacking multiple layers.

During training and evaluation of the baselines, we generated hard negative samples with a replacement ratio of $r=0.2$^1^ and applied binary cross-entropy loss to distinguish positive and negative samples (except for AHP, which used its adversarial loss).

**Table 7:** Optimal hyperparameters for HypergraphConv + HyHG

Hyperparameter	Neurology	Immunology	Embryology
Hyperedge embedding dimension (*d*)	64	64	64
Batch size	64	64	64
Weight decay	$3 \times 10^{-4}$	$2 \times 10^{-4}$	$3 \times 10^{-4}$
Learning rate	$1 \times 10^{-4}$	$1 \times 10^{-4}$	$1 \times 10^{-4}$
Masked self-attention heads (*H*)	4	4	4

*Training Details.* To improve interpretability and reduce noise, we discard hyperedges with fewer than 5 or more than 30 concepts—small ones are trivial, while large ones aggregate loosely related terms, hindering higher-order reasoning. This filtering results in a mean and standard deviation of $\sim$11 and $\sim$6 concepts per hyperedge, respectively. HyHG is trained using the Adam optimizer on mini-batches of hyperedges and their trails of ideas, optimized via our proposed temporal hypergraph contrastive learning framework. We fix $\beta =0.3$, $\alpha =0.3$, $d_{\text {time}}=16$, and $\text {Margin}=1.0$ across all datasets, while other hyperparameters are manually tuned per HyHG configuration and dataset. Table 7 reports the optimal hyperparameters for the best-performing setup (HypergraphConv + HyHG).

## Empirical support for theoretical claims

**Table 8:** AUC / AP of varying node?replacement rates *r*

Model	*r*	Neurology		Immunology		Embryology	
		AUC	AP	AUC	AP	AUC	AP
UniGCN	1.0	0.965	0.943	0.969	0.949	0.973	0.958
	0.5	0.778	0.737	0.858	0.809	0.862	0.822
	0.2	0.607	0.563	0.603	0.560	0.634	0.579
UniGAT	1.0	0.973	0.958	0.979	0.966	0.986	0.978
	0.5	0.783	0.756	0.878	0.843	0.896	0.884
	0.2	0.600	0.560	0.605	0.561	0.537	0.522
Cheshire	1.0	0.999	0.999	0.998	0.998	0.997	0.997
	0.5	0.927	0.926	0.959	0.956	0.954	0.958
	0.2	0.620	0.607	0.659	0.620	0.623	0.606
HypergraphConv	1.0	0.957	0.934	0.967	0.954	0.985	0.977
	0.5	0.813	0.748	0.851	0.811	0.908	0.889
	0.2	0.614	0.567	0.611	0.564	0.629	0.578

### B.1 Empirical support for claim 1

Table 8 shows how varying the concept replacement rate *r* in negative sampling affects HCN performance across Neurology, Immunology, and Embryology. As *r* decreases, both AUC and Average Precision (AP) drop consistently across models and datasets. A lower *r* means fewer concept replacements, making negative hyperedges more similar to their positive counterparts. This increases classification difficulty for the model $f_\theta$, confirming Claim 1: that lower *r* produces harder negatives. Additionally, Table 2 in the main text compares HyHG to AHP, which uses adversarial training for hard negative generation. Despite AHP’s adversarial negative sampling, HyHG outperforms it under our evaluation setup, further validating Claim 1.

**Table 9:** Euclidean distance of hyperedge embeddings to their historical context, with and without Time-anchored Contrastive Loss (TACL)

	**Neuro.**		**Immun.**		**Embryo.**	
Method	Neg	Pos	Neg	Pos	Neg	Pos
w/o TACL	4.50	5.34	5.01	5.48	5.86	5.91
w/ TACL	1.10	0.96	1.87	1.16	1.22	0.86

### B.2 Empirical support for claim 2

Table 9 reports Euclidean distances between the historical context embeddings and those of both positive and hard negative hyperedges. Without the time-anchored contrastive loss, hard negatives are closer to historical context than positives, indicating poor temporal discrimination. When the contrastive loss is applied, the embeddings of positive hyperedges become consistently closer to their historical context than the hard negatives, supporting Claim 2. This demonstrates that anchoring in historical context encourages the model to distinguish true hypotheses from deceptive alternatives, even when negatives are generated with low *r*. Overall, these results confirm that the time-anchored contrastive loss enhances the model’s ability to exploit temporal structure, improving its discriminative power in challenging settings.
